# Exploring the Effects of Probiotic Treatment on Urinary and Serum Metabolic Profiles in Healthy Individuals

**DOI:** 10.1021/acs.jproteome.3c00548

**Published:** 2023-11-16

**Authors:** Francesca Di Cesare, Matteo Calgaro, Veronica Ghini, Diletta Francesca Squarzanti, Annachiara De Prisco, Annalisa Visciglia, Paola Zanetta, Roberta Rolla, Paola Savoia, Angela Amoruso, Barbara Azzimonti, Nicola Vitulo, Leonardo Tenori, Claudio Luchinat, Marco Pane

**Affiliations:** † Magnetic Resonance Center (CERM), University of Florence, Via Luigi Sacconi 6, Sesto Fiorentino, Firenze 50019, Italy; ‡ Department of Chemistry “Ugo Schiff”, University of Florence, Via della Lastruccia 3, Sesto Fiorentino 50019, Italy; § Department of Biotechnology, University of Verona, Strada le Grazie, 15, Verona 37134, Italy; ∥ Department of Health Sciences (DiSS), University of Piemonte Orientale (UPO), Via Solaroli, 17, Novara 28100, Italy; ⊥ Center for Translational Research on Autoimmune and Allergic Diseases (CAAD), Department of Health Sciences (DiSS), University of Piemonte Orientale (UPO), Corso Trieste, 15, Novara 28100, Italy; # Probiotical Research Srl, Via Enrico Mattei, 3, Novara 28100, Italy; ¶ Consorzio Interuniversitario Risonanze Magnetiche MetalloProteine (CIRMMP), Via Luigi Sacconi 6, Sesto Fiorentino, Firenze 50019, Italy; ∇ Giotto Biotech S.r.l., Via Madonna del Piano, 6, Sesto Fiorentino, Firenze 50019, Italy

**Keywords:** NMR-based metabonomics, probiotics, metabolites, phenotype

## Abstract

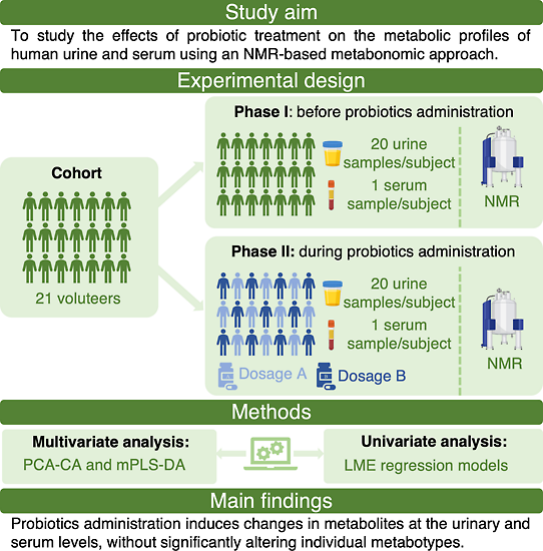

Probiotics are live microorganisms that confer health benefits when administered in adequate amounts. They are used to promote gut health and alleviate various disorders. Recently, there has been an increasing interest in the potential effects of probiotics on human physiology. In the presented study, the effects of probiotic treatment on the metabolic profiles of human urine and serum using a nuclear magnetic resonance (NMR)-based metabonomic approach were investigated. Twenty-one healthy volunteers were enrolled in the study, and they received two different dosages of probiotics for 8 weeks. During the study, urine and serum samples were collected from volunteers before and during probiotic supplementation. The results showed that probiotics had a significant impact on the urinary and serum metabolic profiles without altering their phenotypes. This study demonstrated the effects of probiotics in terms of variations of metabolite levels resulting also from the different probiotic posology. Overall, the results suggest that probiotic administration may affect both urine and serum metabolomes, although more research is needed to understand the mechanisms and clinical implications of these effects. NMR-based metabonomic analysis of biofluids is a powerful tool for monitoring host-gut microflora dynamic interaction as well as for assessing the individual response to probiotic treatment.

## Introduction

Several numbers of microorganisms, approximately 1.3 times more than host cells, exist and coexist in the human gastrointestinal tract, and they directly maintain and modulate the metabolic and molecular balance of the gut environment.^1−4^ It is demonstrated that the highly complex net of microorganisms that compose the gut microbiota, thanks to the production of specific antimicrobial proteins and the change of redox status, pH, and nutrient distribution, prevents the adhesion, proliferation, prevarication, and virulence of exogenous and endogenous microorganisms, determining the fortification of the host’s gut immunity barrier.^5−8^ Moreover, gut microorganisms are responsible for the regulation of many important human physiological pathways, including those involved in the synthesis of proinflammatory cytokine,^9,10^ reactive oxygen compounds,^11,12^ enzymes able to digest polysaccharide,^13,14^ and the production of vitamin K, and most of the water-soluble B vitamins, such as biotin, cobalamin, folates, nicotinic acid, pantothenic acid, pyridoxine, riboflavin, and thiamine^15−17^ in humans. The human microbiota, in this way, contributes to the host’s metabolism and physiology.

In this scenario, it is possible to define the host-microbiome interactions as generative, from a genetic and a metabolic point of view, of a superorganism, called holobiont,^18,19^ and the individual phenotypes are seen as direct results of these complex and dynamic interactions.^20−23^ The gut-microbiota composition regularly experiences changes in terms of structure and function. These changes could be dependent on physiological aspects (*i.e.*, age, sex, BMI, etc.) and lifestyle and clinical aspects (*i.e.*, diet, medical condition, drug treatments, etc.).^24−26^ When the microflora balance is definitely altered, human well-being could be compromised, driving several pathophysiological alterations.^27−30^ In this light, to preserve and promote the healthy interactions between host and microbiota, probiotics, defined as “living microorganisms, which when administered in adequate amounts confer health benefits on the host”,^31−33^ are increasingly used as dietary supplements or functional food for improving balanced microbial communities, for the suppression of potential pathogens, for the immunomodulation, and the stimulation of epithelial cell proliferation and fortification.^34^ Currently, high-throughput metagenomic studies have been widely conducted to achieve deep characterization of the gut microbiome strain-level composition after the probiotic treatments,^35−39^ but the metabolic interaction between probiotics and their host remains only partially understood and investigated. Therefore, to better understand the holobiont metabolic interactions in health, changes in the functional and metabolic composition of the gut microbiota should be deeply explored.

In this context, nuclear magnetic resonance (NMR)-based metabonomics represents a powerful technique to investigate the complex molecular mechanisms and the highly interconnected dynamics between the host and the associated microbiota, taking into account the response to probiotic administration, providing crucial information about several metabolites detectable in biological fluids (*i.e.*, serum, plasma, and urine), shedding a deeper light on the metabolic function that the microbiota exerts on human health.^40−43^

Several metabonomic studies on the administration of probiotics in patients in the state of health and disease demonstrated that the microbiota is intrinsically associated with overall health, including gut pathologies in both adults and children, clarifying how much probiotics can influence the healthy microbial community and the physiological functions.^44−48^ In this work, we analyzed and characterized by NMR spectroscopy the metabolic concentration changes in urine and plasma samples of twenty-one healthy adult volunteers, regularly administered with two different probiotic posologies. The probiotic blends were composed of strains of the *Lactiplantibacillus plantarum*, *Lacticaseibacillus rhamnosus*, *Limosilactobacillus fermentum,* and *Bifidobacterium longum* species and the period of the administration was characterized by a total of 8 weeks. As previously demonstrated,^44^ to have a more robust image of the metabolic behavior of the holobiont, a strong point that we proposed in this work is the use of different biofluids (urine and serum) and 20 samples per subject for urine, considered much more related to inherent variability than serum. Univariate and multivariate analyses were used to evaluate the one-dimensional (1D) NMR urine and serum spectra and to evaluate the effect of the probiotics on the human metabolome.

## Materials and Methods

### Study Population

The trial was registered at clinical.trials.gov with the registration number NCT04506385. The study group consists of twenty-one adult healthy volunteers with an overall age range from 24 to 64 years (6 men with a mean age of 52.8 ± 10.8 years and 15 women with a mean age of 40.7 ± 10.8 years), whose demographic characteristics are reported in Table 1.

**Table 1:** Demographic Characteristics of Healthy Adult Volunteers Enrolled in the Study

	all	women	men
*n* (*n*, %)	21	15 (71.4)	6 (28.6)
age (yrs ± SD)	45.9 ± 11.8	40.7 ± 10.8	52.8 ± 10.8
height (m ± SD)	1.70 ± 0.09	1.65 ± 0.05	1.82 ± 0.07
weight (kg ± SD)	66.5 ± 14.7	59.4 ± 8.3	84.3 ± 11.9
BMI (kg/m^2^± SD)	22.7 ± 3.1	21.7 ± 2.8	25.4 ± 2.3

### Study Design

**Figure 1:**
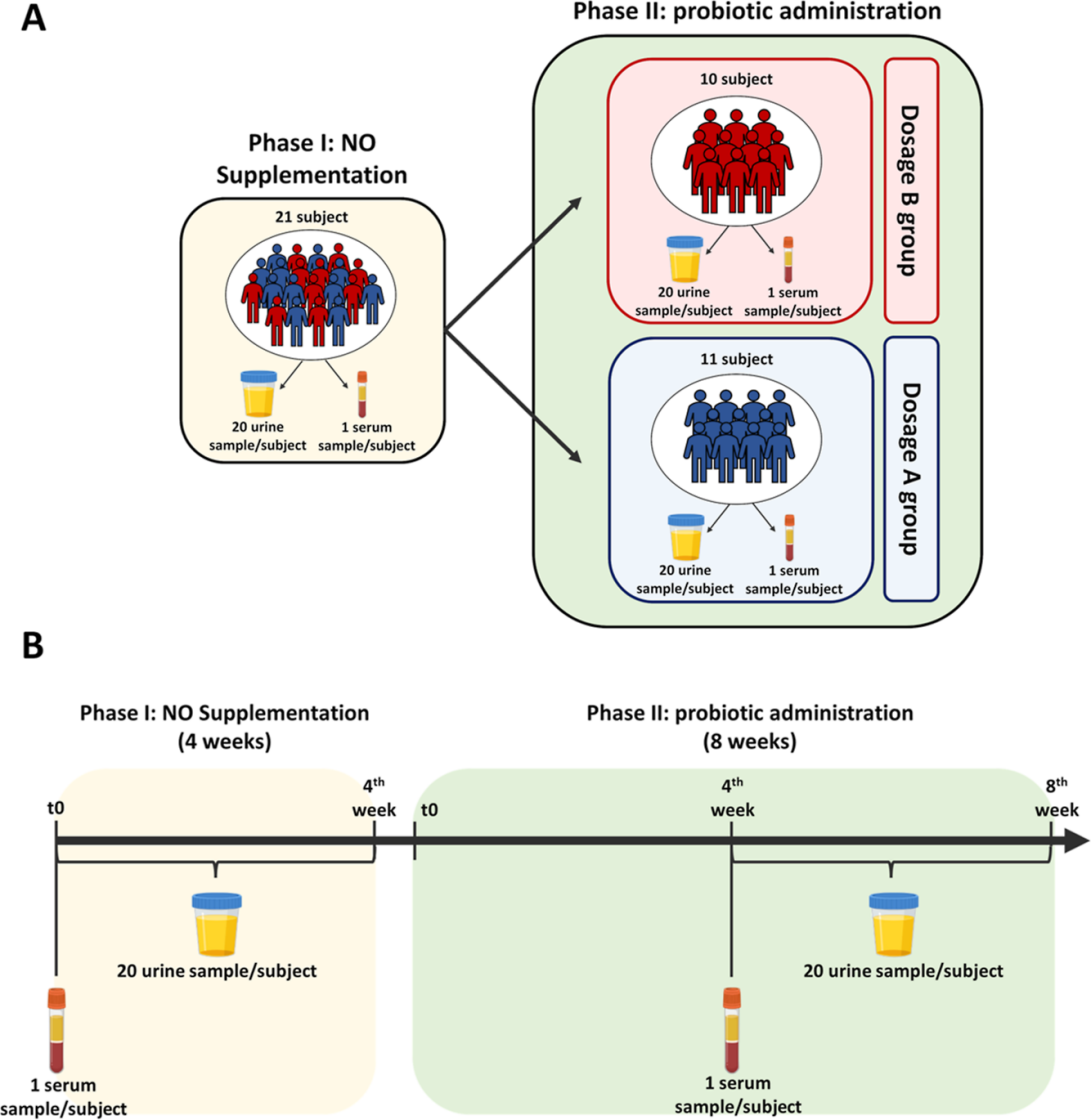
Study design. (A) Experimental scheme of the two phases of the project; (B) temporal scheme of urine and serum sampling in phase I and phase II.

The study was based on two different phases (Figure 1A,B):

Phase I: as performed by Ghini *et al.*,^44^ the first phase of the study was characterized by a period of 4 weeks during which the healthy volunteers did not take any supplementation of probiotics. During this phase, each volunteer collected 20 urine samples (1 sample per day, excluding the weekends and the menstrual cycle days) and proceeded with their usual diet and lifestyle. At the beginning of phase I, a serum sample from each subject was also collected.

Phase II: the second phase of the study was a period of 8 weeks during which the volunteers were administered with a daily dose of probiotics. In this phase, the subjects were randomly divided into two groups, named “dosage A” and “dosage B”. The “dosage A” group (*n* = 11 subjects) added to their usual diet a daily dose of 4 billion of the same probiotic mixtures; the “dosage B” group (*n* = 10 subjects) added to their usual diet a daily dose of 40 billion of probiotic strains. Starting from day 28 of probiotic assumption, each volunteer collected 20 urine samples (1 sample per day, excluding the weekends and the menstrual cycle days) and proceeded with their usual diet and lifestyle. In the fourth week of probiotic assumption of phase II, a serum sample from each subject was also collected.

### Ethical Issues

The study was conducted in accordance with the Declaration of Helsinki (1964) and its later amendments. Informed, written consent was obtained from all participants. Ethical approval (protocol n◦ 294/CE, study n◦ CE 14/20, International Ethics Committee A.O.U. “Maggiore della Carità”, Novara, Italy) was obtained.

### Sample Collection

42 serum and 840 urine samples were collected during the entire course of the study. All serum samples were collected under overnight fasting conditions. For urine, the midstream of the first urine of the morning was collected. The pre-analytical treatment of all the samples followed standard operating procedures (SOPs) to obtain high-quality specimens for metabolomic analysis.^49−52^

Blood samples were collected in serum blood collection tubes without anticoagulants at room temperature. The samples were processed within 2 h of the blood draw. The samples were centrifuged at room temperature for 10 min at 1500*g*, then serum was collected, and the aliquots were transferred into prelabeled cryovials. Urine samples were collected in sterile plastic cups. All the samples were processed within 2 h from the collection; centrifugation at 1000–3000*g* for 5 min at +4 °C was followed by a filtration using 0.20 μm cutoff filters. All the processing procedures are detailed in the paper by Takis *et al.*(42)

After processing, both serum and urine samples were stored at −80 °C until analysis.

### Probiotic Formulations

The commercial probiotic formulation (2.5 g) administered during the study was a blend of the four strains *Lactiplantibacillus plantarum* LP01 (LMG P-21021), *Limosilactobacillus fermentum* LF16 (DSM 26956), *Lacticaseibacillus rhamnosus* LR06 (DSM 21981), and *B. longum* 04 (DSM 23233) all belonging to Probiotical S.P.A. collection. The probiotic strains were blended with maltodextrin to obtain two different cell loads to administer during the study. The clinical formulas had a cell potency measured by a plate count approach of 4 × 10^9^ colony forming units (cfu)/dose or 40 × 10^9^ cfu/dose (Biolab Research Method 014-06). Cell potency of the samples was also measured by flow cytometer (ISO 19344:2015 IDF 232:2015) resulting in values of >4 × 10^9^ active fluorescent units (afu)/dose and >40 × 10^9^ afu/dose. The probiotic formulations were referred to as dosage A (4 × 10^9^ live cells/dose) and dosage B (>40 × 10^9^ live cells/dose) for the lower and higher potency probiotic doses, respectively.

### NMR Sample Preparation

NMR samples were prepared according to SOPs for urine and serum.^41,49^ Frozen samples were thawed at room temperature and shaken before use. A total of 300 μL of each plasma sample was added to 300 μL of a phosphate sodium buffer (70 mM Na_2_HPO_4_; 20% (v/v) ^2^H_2_O; 0.025% (v/v) NaN_3_; 0.8% (w/v) sodium trimethylsilyl [2,2,3,3-^2^H_4_] propionate (TSP) pH 7.4); a total of 750 μL of each urine sample was centrifuged at 14,000*g* for 5 min, and 630 μL of the supernatant was added to 70 μL of a potassium phosphate buffer (1.5 M K_2_HPO_4_, 100% (v/v) ^2^H_2_O, 10 mM sodium trimethylsilyl [2,2,3,3 ^2^H_4_] propionate (TMSP) pH 7.4). The mixtures were homogenized by vortexing for 30 s, and a total of 600 μL of each mixture was transferred into a 5.00 mm NMR tube (Bruker BioSpin, Rheinstetten, Germany) for analysis.

### NMR Analysis and Processing

One-dimensional (1D) ^1^H NMR spectra were acquired using a Bruker 600 MHz spectrometer (Bruker BioSpin s.r.l., Germany) optimized for metabolomic samples, operating at 600.13 MHz and equipped with a 5 mm cryoprobe, an automatic tuning-matching (ATM), and an automatic sample changer. In the NMR probe, the samples were kept for 3 min ahead for temperature equilibration and maintenance. The acquisition temperature used was 300 K for urine and 310 K for serum samples.

According to standard procedures, for each serum sample, three 1D ^1^H NMR spectra were acquired with water peak suppression and different pulse sequences: (i) a standard nuclear Overhauser effect spectroscopy (NOESY)^53^ 1Dpresat (noesygppr1d.comp; Bruker BioSpin) pulse sequence, using 32 scans, 98304 data points, a spectral width of 18,028.846 Hz, an acquisition time of 2.7 s, a relaxation delay of 4 s, and a mixing time of 0.1 s. (ii) A standard Carr–Purcell–Meiboom–Gill (CPMG)^54^ (cpmgpr1d.comp; Bruker BioSpin) pulse sequence, using 32 scans, 73728 data points, a spectral width of 12019.230 Hz, and a relaxation delay of 4 s. (iii) A standard diffusion-edited (ledbgppr2s1d.comp; Bruker BioSpin) pulse sequence, using 32 scans, 98304 data points, a spectral width of 18028.846 Hz, and a relaxation delay of 4 s.

For each urine sample, only 1D ^1^H NMR spectra were acquired with water peak suppression and a standard NOESY pulse sequence using 64 scans, 65536 data points, a spectral width of 12019.230 Hz, an acquisition time of 2.7 s, a relaxation delay of 4 s, and a mixing time of 0.1 s. Samples collected from the different subjects were mixed and acquired in a totally random order to avoid any batch effects. All the NMR spectra were automatically corrected for phase and baseline distortions and calibrated to the reference signal of TMSP at δ 0.00 ppm and to the glucose doublet at δ 5.24 ppm for urine and serum, respectively, using TopSpin 3.6.2 (Bruker BioSpin Gmbh, Germany). Each spectrum in the range 0.2–10.0 ppm was segmented into 0.02 ppm chemical shift bins, and the corresponding areas were integrated using AssureNMR software (Bruker BioSpin s.r.l., Germany); the region between 6.0 and 4.5 ppm containing residual water signal was excluded. For urine samples, normalization was applied to the obtained bins to minimize dilution effects caused, for example, by variation in fluid intake; the area of each bin was normalized using probabilistic quotient normalization (PQN).^55^ Unlike urine, the serum is not affected by dilution effects, and solute concentrations are tightly controlled; thus, for serum spectra, any normalization method was applied.

### Metabolite Assignment and Quantification

28 metabolites in serum samples and 38 metabolites in urine samples were correctly assigned in all spectra using a ^1^H NMR spectra library of pure organic compounds (BBIOREFCODE, Bruker BioSpin), public databases, as Human Metabolome Database,^56^ storing reference ^1^H NMR spectra of metabolites, and using information available in the literature. Matching between new NMR data and databases was performed using AssureNMR and AMIX software (Bruker BioSpin s.r.l., Germany). The quantification of the assigned metabolites was directly performed by integrating the signals in the spectra in a defined spectral range using a house-developed tool.

For completeness, the metabolites correctly assigned and quantified in both urine and serum samples are presented in Supporting Information Table S1.

### Statistical Analysis

#### Multivariate Analysis

First, the principal component analysis (PCA)–canonical analysis (CA)^57,58^ was performed to increase the supervised data visualization, data space reduction, cluster detections, and group discrimination.

To obtain pairwise comparisons, before and during the treatment, multilevel partial least square discriminant analysis (mPLS-DA)^59^ was employed. For all classification models, the accuracy, sensitivity, and specificity were calculated according to the standard definition. Moreover, the results were validated using the Monte Carlo cross-validation algorithm (MCCV).^60^ Using this approach, the original data set was randomly split into a training set, containing 80% of the data, which was used to assess the test set, containing the remaining 20% of data. This procedure was repeated *k* = 100 times.

#### Univariate Analysis

To study the metabolite trends and their relationship with the treatment, a mixed-effect linear regression framework was employed for each metabolite. Using a simplified notation, the full model for the log-quantification of a generic urine metabolite was specified as follows
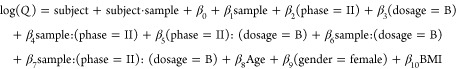
where:

- log(*Q*) is the dependent variable of the model, *i.e.*, the log-quantification of a generic metabolite;
- subject + subject·sample is the random part of the model: each subject has a random intercept and slope, hence, for each subject, the trend in consecutive samples defined by the variable sample (numerical, from 1 to 40, one for each subject’s measurement) could be different;
- β_0_ is the fixed intercept;
- β_1_ to β_7_ are the coefficients for the sample number, treatment dosage (categorical, A or B), phase (categorical, I for samples collected before the probiotic supplementation, II for samples collected during the probiotic supplementation), and their interactions;
- β_8_ to β_10_ are the coefficients for age, gender, and BMI which were included in the models as they are considered possible confounding variables;
- the reference level for this model is represented by a male subject, belonging to the dosage A group, before the treatment.

To obtain a reduced model, which is more parsimonious than the full model, but with a comparable ability to describe the data variability, a stepwise model selection procedure was used.

Similarly, the full model for the log-quantification of a generic serum metabolite was specified as follows
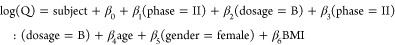
where:

- log(*Q*) is the dependent variable of the model, i.e., the log-quantification of a generic metabolite;
- subject is the random intercept for each subject;
- β_0_ is the fixed intercept;
- β_1_ to β_3_ are the coefficients for the phase of the measurement (categorical, I if the measurement belongs to the pretreatment period, II otherwise), treatment dosage (categorical, A or B), and their interactions;
- β_4_ to β_6_ are the coefficients for age, gender, and BMI which were included in the models as they are considered possible confounding variables;
- the reference level for this model is represented by a male subject, belonging to the dosage A group before the treatment intake starts.

Once the models had been estimated, linear combinations of the parameters were used, and 90% confidence intervals were computed to describe trends (*i.e.*, average differences between subsequent measurements) and average differences between phases for each metabolic log-quantification (see Supporting Information Methods for more details).

### Software

All calculations were performed in the R (v 4.3.2) statistical environment. All plots were obtained using the “ggplot2”^61^ R package. The multivariate analyses were carried out using R software developed in-house. The mixed-effects models were estimated using the “nlme”^62,63^ R package.

## Results

### Effect of Dosage-dependent Probiotic Administration on Urinary Metabolic Human Phenotype

To characterize the urinary individual metabolic phenotype of the healthy subjects, and to investigate the effect of the probiotic and the dosage-specific probiotic assumption on the metabolic profile, the principal component analysis–canonical analysis–K-nearest neighbors (PCA–CA–KNN) statistical model, also used in previous studies conducted by our research group, was performed.^44^

**Figure 2:**
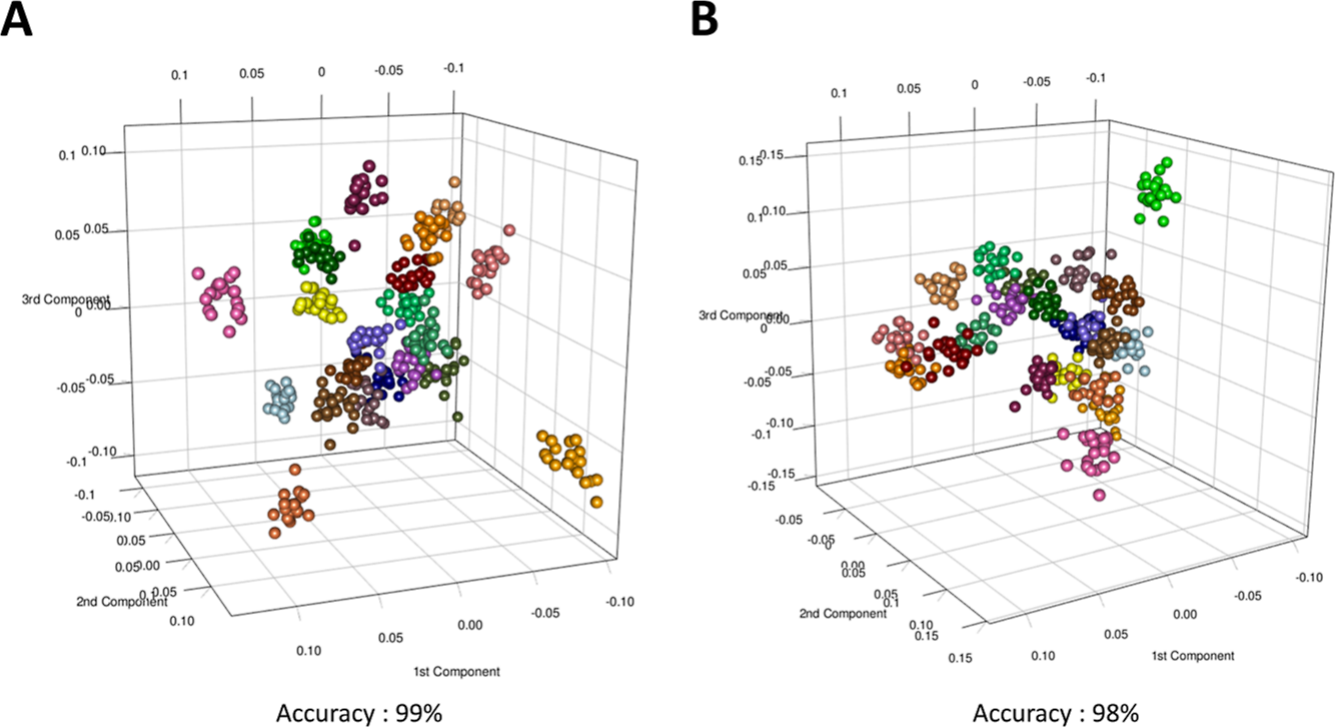
Urinary subject-specific metabolic phenotype discrimination in (A) phase I; (B) phase II. Each color in the PCA–CA score plot represents a different healthy subject. At the bottom of the score plot, the accuracy of the model, expressed in percentage, is also reported.

As expected,^21,44^ considering all urine samples collected before the administration of the probiotics at the baseline reference (phase I), the individual discrimination was almost perfect, with an accuracy value of 99% (Figure 2A). During the probiotic treatment, individual discrimination decreases by 1% passing from 99% in phase I to 98% in phase II (Figure 2B).

**Figure 3:**
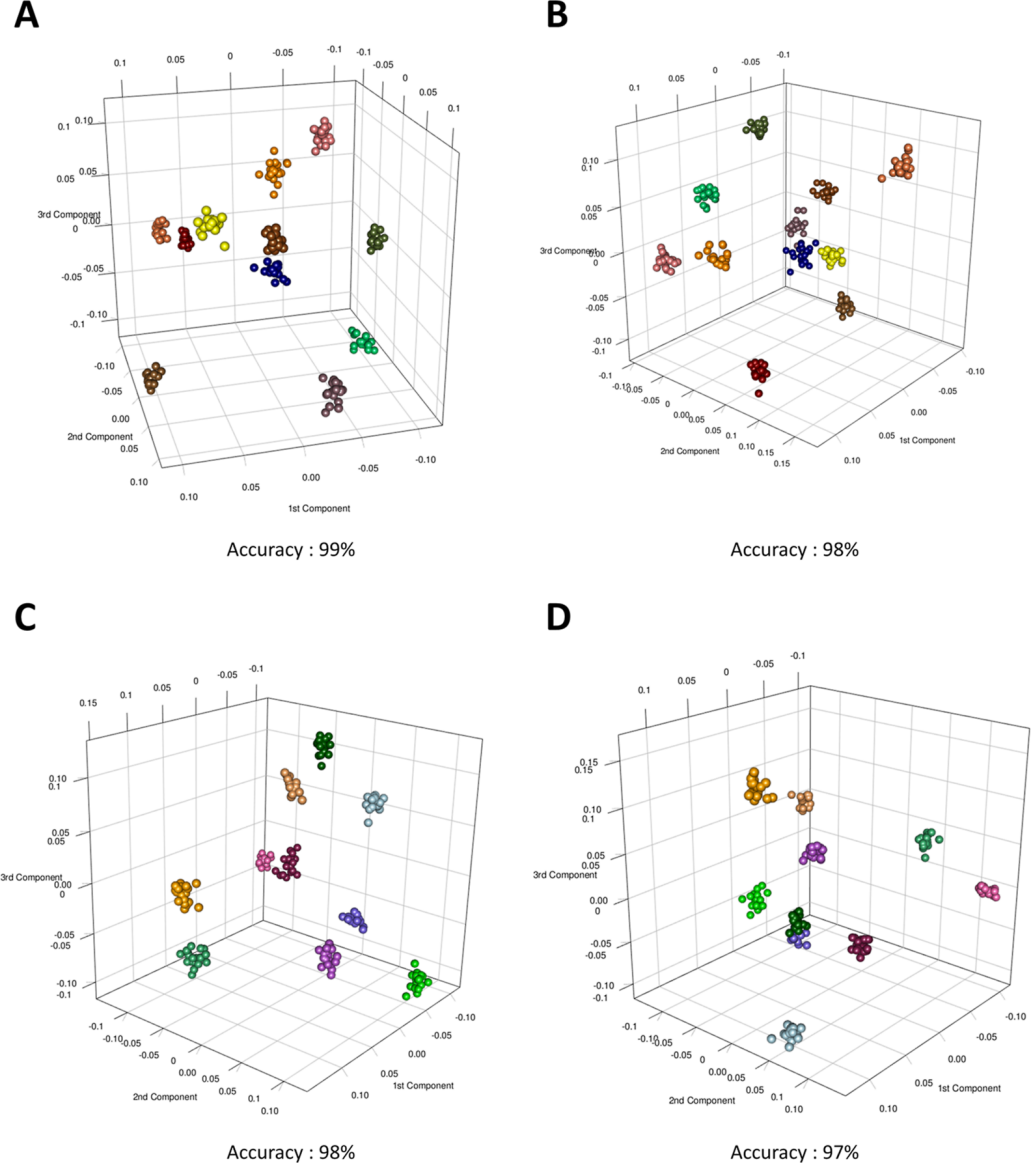
Discrimination of urinary dosage-dependent subject-specific metabolic phenotype in (A) phase I in subjects administered with the dosage A of probiotics; (B) phase II in subjects administered with the dosage A of probiotics; (C) phase I in subjects administered with the dosage B of probiotics; and (D) phase II in subjects administered with the dosage B of probiotics. Each color in the PCA–CA score plots represents a different healthy subject. At the bottom of each score plot, the accuracy of the model, expressed in percentage, is also reported.

Performing the same analysis on the dosage-specific groups separately, we observed the same behavior. In particular, the subjects treated with the dosage A of probiotics showed, in phase I (Figure 3A), an accuracy discrimination of 99% and, during the treatment, an accuracy discrimination of 98% (Figure 3B). The subjects treated with the dosage B of probiotics passed from an individual discrimination accuracy of 98% before the treatment (Figure 3C) to 97% during the treatment (Figure 3D).

### Effect of Different Dosages of Probiotics on the Urinary Metabolome

**Figure 4:**
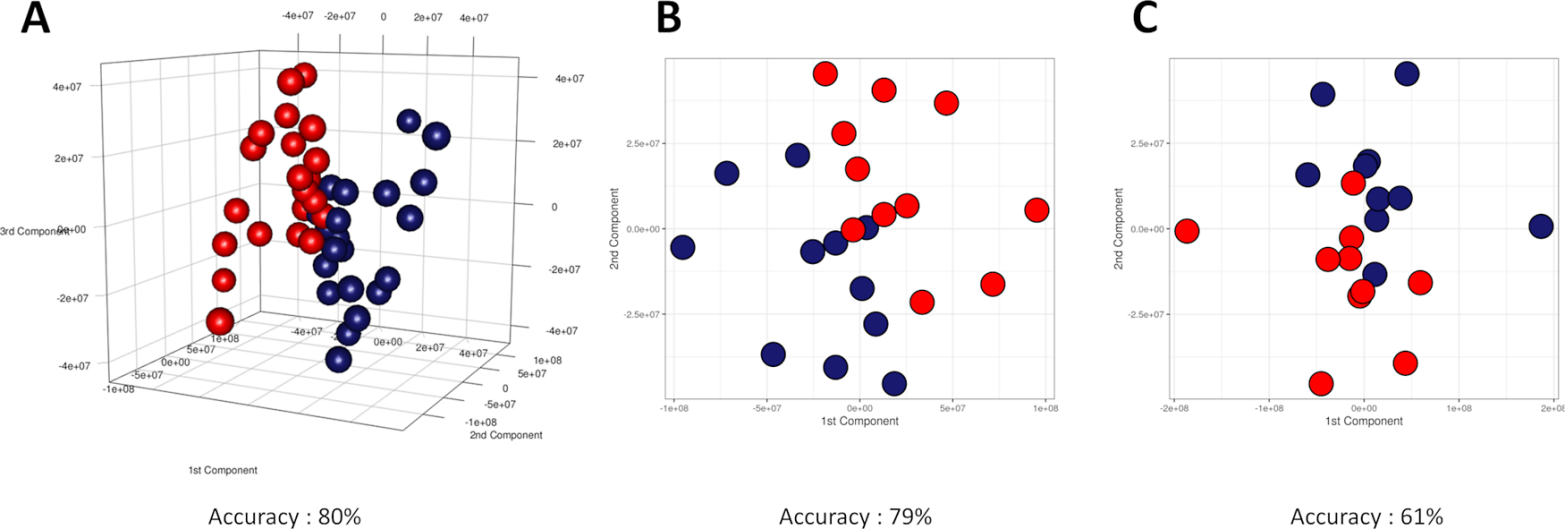
Score plots of M-PLS discrimination between urine samples collected (A) for all subjects during phase I (blue dots) and phase II (red dots); (B) for subjects administered with dosage A of probiotics during phase I (blue dots) and phase II (red dots); and (C) for subjects administered with dosage B of probiotics during phase I (blue dots) and phase II (red dots). Discrimination accuracy values for the three pairwise comparisons were also reported. The median spectrum of each subject at every phase was calculated and used to build the MPLS models.

To highlight a potential global effect and a potential dosage-dependent effect of the probiotic assumption, minimizing the intraindividual variability, the entire urine spectra collected during the two treatment phases were compared using M-PLS analysis (Figure 4). Using this statistical approach, we evaluated how much the urinary profile changes after the introduction of an exogenous set of microorganisms.

We observed moderate discrimination (80%) and good separation between urine metabolome before and during treatment, considering the entire cohort of healthy volunteers (Figure 4A).

Investigating a dosage-dependent effect, we interestingly and unexpectedly observed that the subjects treated with a lower dose of probiotics tended to have a discrimination accuracy higher than that of the subjects treated with a higher dose of probiotics (Figure 4B,C); more precisely 79% accuracy for the first group and 61% for the second group.

To describe metabolic variations, a mixed-effect regression model was implemented for each urinary metabolite (for more details, see Materials and Methods section, Supporting Information Methods 1.1 and Figure S1A).

First, the presence of trends (*i.e.*, average differences between subsequent measurements) in log-quantification levels was tested. Considering each phase and dosage group separately, estimated log-quantification values between consecutive samples were considered. Positive differences represent ascending trends, while negative differences describe descending trends. Regarding statistically significant ascending trends, we observed formate for both dosage groups and phases, acetoacetic acid, sugar unknown (unk), and glucose for both dosage groups during phase II, hippurate for the dosage A group during phase I, and acetone and 2-hydroxyisobutyric acid for the dosage B group during phase I. Instead, we observed decreasing trends for phenylacetylglutamine, sugar unk, glucose, 4-hydroxyphenylacetate, and acetoacetic acid for both dosage groups during phase I, trimethylamine-*N*-oxide and lysine for both dosage groups during phase II, dTTP and creatinine for the dosage A group during both phases, acetone for the dosage B group during phase II, and isoleucine for the dosage A group during phase I (Table 2 and Figure S3).

**Table 2:** Significant Trends (*i.e.*, Average Difference between Subsequent Measurements) by Phase and Dosage Group for Urinary Metabolite Log-quantifications (with 90% Confidence Intervals), Adjusted for other Variables^a^

metabolite	estimate	lower	upper	phase	dosage
hippurate	0.0178	0.0085	0.0271	I	A
2-hydroxyisobutyric acid	0.0080	0.0049	0.0112	I	B
Acetone	0.0065	0.0013	0.0116	I	B
phenylacetylglutamine	–0.0031	–0.0059	–0.0003	I	A and B
sugar unk (ppm range = 5.218–5.200)	–0.0032	–0.0062	–0.0002	I	A and B
isoleucine	–0.0034	–0.0058	–0.0009	I	A
4-hydroxyphenylacetate	–0.0037	–0.0074	←0.0001	I	A and B
glucose	–0.0057	–0.0101	–0.0014	I	A and B
acetoacetic acid	–0.0066	–0.0120	–0.0012	I	A and B
acetoacetic acid	0.0067	0.0013	0.0121	II	A and B
glucose	0.0044	0.0001	0.0088	II	A and B
sugar unk (ppm range = 5.218–5.200)	0.0032	0.0002	0.0061	II	A and B
lysine	–0.0024	–0.0045	–0.0004	II	A and B
acetone	–0.0076	–0.0127	–0.0025	II	B
TMAO	–0.0151	–0.0241	–0.0062	II	A and B
formate	0.0046	0.0009	0.0082	I and II	A and B
dTTP	–0.0029	–0.0050	–0.0008	I and II	A
creatinine	–0.0034	–0.0055	–0.0013	I and II	A

a The positive estimate value indicates an increasing significant trend and the negative estimate value indicates a decreasing significant trend.

Finally, estimated log-quantification levels were tested for differences between phase II and phase I (net of other variables by dosage group). To accomplish this task, the estimated average log-quantification values for phase II and phase I were compared. 3-Hydroxyisobutyric acid, 4-hydroxyphenylacetate, and glucose were decreased in phase II for both dosage groups, valine and isoleucine were significantly decreased only for the dosage A group, allantoin and unknow 4 (unk4) (ppm range = 5.410–5.400) were decreased only for the dosage B group, while tartrate was significantly increased for the dosage A group (Table 3 and Figure S4). For the sake of completeness, we performed the same analyses assuming the equality of the dosage-specific effects over time in the entire cohort of healthy volunteers. Overall, the results remained stable and can be found in the Supporting Information, Tables S2, S3 and Figures S5, S6 (for more details, see Supporting Information Methods, Urine Metabolites—Unique Dosage, and Figure S1B).

**Table 3:** Estimated Average Differences between Phase II and Phase I for Urinary Metabolite Log-quantifications (90% Confidence Intervals), Adjusted for other Variables

metabolites	estimate	lower	upper	dosage
tartrate	0.402	0.241	0.563	A
valine	–0.036	–0.066	–0.006	A
3-hydroxyisobutyric acid	–0.040	–0.070	–0.010	A and B
4-hydroxyphenylacetate	–0.050	–0.083	–0.016	A and B
isoleucine	–0.053	–0.082	–0.023	A
unk4 (ppm range = 5.410–5.400, singlet)	–0.084	–0.132	–0.036	B
allantoin	–0.087	–0.140	–0.035	B
glucose	–0.127	–0.174	–0.080	A and B

### Effect of Different Doses of Probiotics on Serum Metabolome

To evaluate the overall effect of probiotics on serum samples, ^1^H NMR serum spectra, collected during both phases, were compared using the same statistical approach as that performed on urine: M-PLS analysis. As reported before, a multilevel approach could be useful for reducing intraindividual variability.

**Figure 5:**
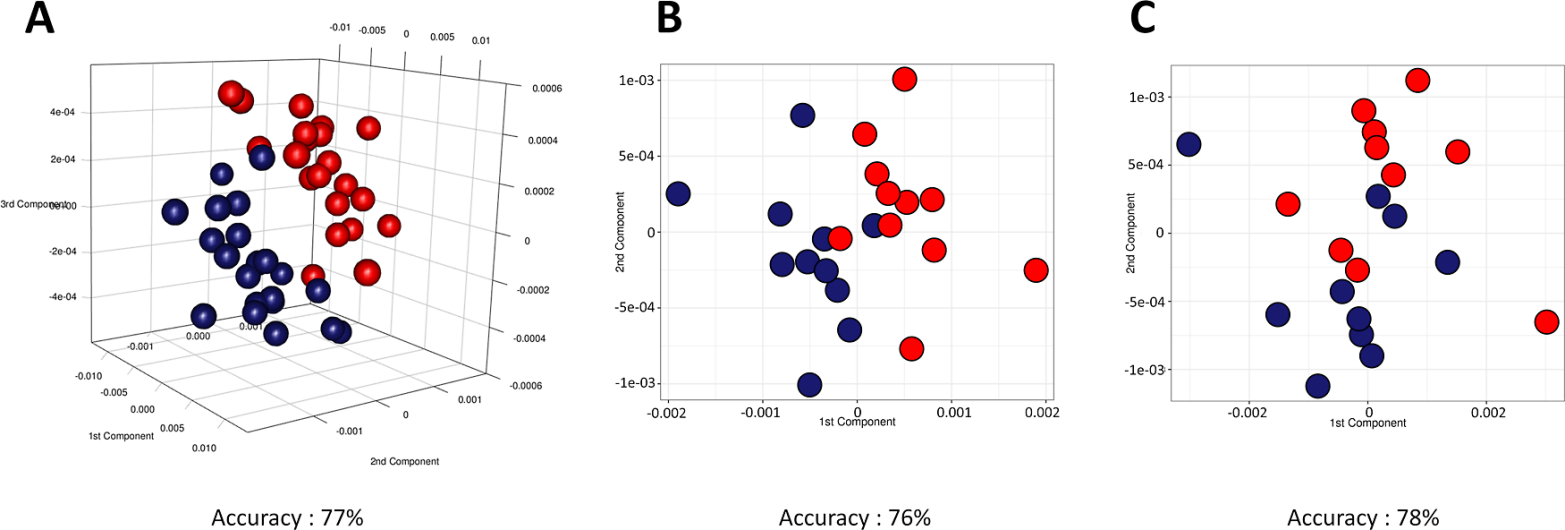
Score plots of M-PLS discrimination between serum samples collected (A) for all subjects during phase I (blue dots) and phase II (red dots); (B) for subjects administered with dosage A of probiotics during phase I (blue dots) and phase II (red dots); and (C) for subjects administered with dosage B of probiotics during phase I (blue dots) and phase II (red dots). Discrimination accuracy values for the three pairwise comparisons were also reported.

We observed fair discrimination (77%) and good separation between serum metabolic profiles before and after treatment, considering the entire cohort of healthy volunteers (Figure 5A).

Investigating the potential dosage-dependent effect, as expected but in contrast with what we assessed in urine samples, we observed that the subjects treated with a lower dose of probiotics tend to have a discrimination accuracy (76%) comparable to that evaluated in the subjects treated with a higher dose of probiotics (78%) (Figure 5B,C).

Similar to the analysis of metabolic variations in urine, a simple mixed-effects regression model was implemented for each serum metabolite (see Materials and Methods section, Supporting Information Methods, Serum Metabolites, and Figure S2).

Log-quantification levels were tested for differences between phase II and phase I (in the absence of other variables). Acetone and pyruvate were significantly increased in phase II for both dosage groups, while histidine, glutamine, creatine, creatinine, acetate, and citrate 1 (ppm range = 2.559–2.545) were significantly decreased (Table 4). The results remain stable not separating the two dosage-specific subcohorts (for more details, see Figure S7).

**Table 4:** Average Differences between Phase II and Phase I for Serum Metabolites Log-quantifications (with 90% Confidence Intervals) Adjusted for other Variables

metabolites	estimate	lower	upper
acetone	0.589	0.478	0.699
pyruvate	0.144	0.042	0.246
histidine	–0.038	–0.065	–0.010
glutamine	–0.048	–0.086	–0.009
creatine	–0.059	–0.116	–0.003
creatinine	–0.066	–0.114	–0.019
acetate	–0.077	–0.131	–0.022
citrate 1 (ppm range = 2.559–2.545, singlet 1)	–0.127	–0.197	–0.058

## Discussion

This study demonstrates that a probiotic administration can lead to changes in metabolites at urinary and serum system levels without significantly altering the individual metabotypes. Our study also demonstrates the paramount role of having access to multiple and prolonged collections of samples in the pretreatment condition to define a reliable baseline.

It is currently well recognized that gut microbiota can produce a wide range of metabolites by human endogenous or exogenous factors (*e.g.*, food compounds) and some of them are exclusive of bacterial origin with a key role in host-microbiota cross-talk.^64^ These metabolic changes driven by bacteria have been documented at fecal, urine, and serum levels.^44,65−68^ Dietary interventions are emerging as a strategy to reshaping and modulate not only the gut microbiota composition, but even their metabolomes, with concomitant positive effects on the hosts.^69^ Similarly, it is demonstrated that the administration of exogenous beneficial bacteria in a close pre-existent ecosystem generates metabolic mutualistic benefits for both the microbial community and the host.^70,71^

In this work, we observed that the administration of probiotics reduces the urinary individual discrimination accuracy by 1%, suggesting that probiotics lead to greater similarity in the metabolic host-microbiome cross-talk.^44^ Although present, we are not able to consider this highlighted effect statistically robust (Figures 2 and 3).

Reducing the intraindividual variability, we determined the overall effect of probiotics on urine subject-specific metabonomic profile. In particular, the urine profiles related to the baseline period were discriminated from the urine profiles collected during the administration of the probiotics with an accuracy of 80%. The same approach was also conducted by dividing our cohort into dosage-dependent groups. Interestingly, a greater overall effect of the noninvasive treatment on the dosage A group (accuracy = 79%) compared with the dosage B group (accuracy = 61%) was recorded (Figure 4). The same approach was also performed on serum samples. The overall effect of probiotic assumption on serum metabonomic profiles was about 77%, considering the entire cohort of study and the two dosage-dependent subgroups (Figure 5). The molecular mechanisms by which these different dosage-dependent effects are generated in metabolomes are still not clear and need to be more deeply investigated; in this context, a complementary intestinal microbiome analysis might shed more insights into the dynamics of metabolic variation.^72^

Overall, statistically significant differences were ascribable to metabolites related to mainly carbohydrates and amino acid metabolism and to bacteria-derived metabolites.

Due to the repeated number of urine samples, the potential ascending and/or descending trends in terms of variation of the levels of the log-quantification metabolites were also analyzed, considering each phase and dosage group separately. In particular, we observed that hippurate in the dosage A group, 2-hydroxyisobutyric acid and acetone in the dosage B group, and 4-hydroxyphenylacetate in both dosage-dependent groups tend to increase in phase I, while isoleucine in the dosage A group, and phenylacetylglutamine, sugar unknown (ppm range = 5.128–5.200), glucose, and acetoacetic acid in both dependent dosage-groups tend to decrease during phase I, suggesting that these variations, not related to the probiotic assumption, are attributable to the diet and lifestyle conducted by the subjects considered in the study. After the probiotic assumption (phase II), we observed an ascendent trend for acetoacetic acid, glucose, and sugar unknown (ppm range = 5.128–5.200) in both dosage-dependent groups. At the same time, formate shows an ascending trend before (phase I) and during the treatment (phase II), and dTTP and creatinine present a descending trend in both phases. It is interesting to note the trend change observed for acetoacetic acid, glucose, and sugar unknown (ppm range = 5.128–5.200) by comparing the results obtained by analyzing separately phase I with phase II (Table 2).

To better understand this phenomenon, the metabolic variations in urine samples were evaluated, taking into account the differences between the two phases. We observed that glucose tends to decrease. This result, compared with the previous ones, suggests that this specific metabolite, although significant, has variations that cannot be totally attributed to the assumption of the probiotic, but it may depend on a set of interconnected causes (*i.e.*, diet, lifestyle, ..., response to the probiotic treatment) which globally determine this metabolic behavior. In contrast, significant changes, potentially attributable to the effect of the probiotic on the urinary metabolome, were observed in 7 out of 38 assigned metabolites (Table 3).

Particularly, 3-hydroxyisobutyric acid and 4-hydroxyphenylacetate significantly decreased during the probiotic treatment in both dosage-dependent groups. It is interesting the role played by 4-hydroxyphenylacetate, an intermediate of tyrosine metabolism, in humans and in microbes. In the human gut, the amino acids (AAs)—that are not digested and absorbed—can be metabolized by the gut microbiota to form the 3- and 4-hydroxyphenylacetate organic acids. Higher levels of these compounds are considered markers to reflect protein malabsorption or dysbiosis.^73−76^ The reduction of urinary 4-hydroxyphenylacetate, highlighted in our study, corroborates the hypothesis of the potentiality of probiotics to rebalance the pre-existent gut microflora, ensuring an improvement in the molecules and AAs homeostasis, necessary for human well-being.

In the dosage A-dependent group only, we observed a significant decrease in valine and isoleucine concentration, while, in the dosage B-dependent group only, we observed a decrease in allantoin and unknown 4 (unk4) (ppm range = 5.410–5.400). The branched-chain AAs (BCAAs), in particular, valine and isoleucine, are essential nutrients with important roles in protein synthesis in humans. The gut microbiota is a major source of circulating BCAAs through biosynthesis and absorption modification, but elevated levels of these circulating molecules are associated with metabolic disorders (*i.e.*, type 1 and 2 diabetes).^77^ The same behavior is observed for 3-hydroxyisobutyric acid. The global reduction of BCAAs and of 3-hydroxyisobutyric acid suggests the potential role of these probiotics in promoting a balanced metabolism (reabsorption and/or modification) of AAs.^77,78^

Urinary allantoin, an end product of purine metabolism, is normally present in urine and is formed from uric acid through reactions with oxidative species. The increase in terms of concentration of this molecule is directly associated with a systemic increase in oxidative stress.^79,80^ In this perspective, one of the beneficial effects ascribed to probiotic assumption is the capability to reduce oxidative stress.^44,81−83^ Accordingly, the administration of the probiotic blend tested in this study might contribute to mitigating the host’s oxidative stress as suggested by the reduction of allantoin biomarkers in urine.^84^

The NMR-spectra region characterized by ppm range from 5.410 to 5.400 is related to sugars (*i.e.*, sucrose, maltose, etc.). It is well known that the presence of sugars in the urine indicates an alteration of their metabolism.^85^ Although the concentration of this sugar in phase I is in the normal range, as a result of the probiotic, we notice a decrease, suggesting that the overall rebalancing of the intestinal microbiota might also play a fundamental role in improving the metabolism of sugars.^86^

Lastly, tartaric acid significantly increases in the dosage A-dependent group. The biological and molecular significance linked to the increase of this compound is still to be discovered.^87^

Considering the serum samples, the levels of the log-quantification metabolites were tested for differences between phase II and phase I. In this case, acetone and pyruvate were significantly increased in phase II, while histidine, glutamine, creatine, creatinine, acetate, and citrate (ppm range = 2.559–2.545) were significantly decreased, independently from the dosage-dependent groups (Table 4). It is demonstrated that the glycolysis impairment can cause a lowering of pyruvate and, also, lactate levels, and an increase of glucose in serum, in particular in diabetic and celiac patients.^44,86,88,89^ The increase of pyruvate, along with the reduction in the level of metabolites with ppm range from 5.410 to 5.400 (*i.e.*, sucrose, maltose, etc.) would corroborate the contribution of the metabolism of exogenous probiotics in sugars metabolism.

During the probiotic assumption, we also observed an increase in acetone levels. As known, the ketone bodies, in particular acetone, are generated as a byproduct of the fat metabolism process.^90−92^*Lactobacilli* tend to increase the metabolic activity of pathways involved in lipid degradation, determining a remodeling in terms of levels of circulating ketone bodies.^93−95^

It is demonstrated that creatine and creatinine are also associated with mitochondrial muscle respiration, playing a regulation role in adipose tissue metabolism;^96,97^ in particular, we can highlight that a potential effect of the probiotics on the protein metabolism is observed even at serum level.^98,99^

The decrease of histidine could be directly related to bacterial fermentation; in fact, the gut microbiota converts histidine into an immunoregulatory signal, histamine, able to suppress pro-inflammatory tumor necrosis factor (TNF) production that could generate several local and/or systemic diseases.^78,100^

It is also known that the gut microbiota utilizes glutamine as a nitrogen source for optimal survival and growth. Alteration in microbiota composition can profoundly influence glutamine metabolism, determining metabolic alteration in pathologies such as fibromyalgia.^101,102^ Probiotic treatments, especially mixtures of *Lactobacilli*, are also used in promoting healthy kidney function; in particular, it was observed in the literature that exogenous microorganisms reduce the overall blood creatinine concentration, which is one of the most relevant biomarkers of chronic kidney disease.^103,104^

## Conclusions

This NMR-based metabonomics study demonstrates that probiotic administration can induce changes in metabolites at the urinary and serum system levels without significantly altering the individual metabotypes. Using multiple biofluids and prolonged sample collections, we established a reliable baseline, allowing for a more robust analysis. The study also highlights the role of bacterial-origin metabolites in host-microbiota cross-talk and the potential for dietary interventions to reshape and modulate the gut microbiota composition and metabolome for positive effects on hosts. Interestingly, this study showed a greater overall effect of the noninvasive treatment on the dosage A group compared to the dosage B group. The molecular mechanisms underlying these effects are still unclear and require further investigation. Statistically significant differences were observed in metabolites related to carbohydrates and amino acid metabolism as well as bacteria-derived metabolites. The results suggest that changes in metabolite levels related to diet and lifestyle were not associated with probiotic intake. Conversely, the metabolites altered by probiotic administration may offer insights into the metabolic function of microbiota in human health. While our study primarily focused on metabonomics, specifically investigating variations in terms of metabolite concentrations resulting from probiotic supplementation, we recognize the importance of translating these findings into clinical contexts. In a more holistic approach, future studies not only should investigate the associations among metabolic changes and specific clinical outcomes (*i.e.*, inflammation processes, immune function, cognitive and mood-related improvements, etc.), but also integrate metagenomic information to obtain a more comprehensive picture of the intricate relationships between probiotics, host metabolism, and health parameters.

## Supporting Information Available

Supplementary methods and Supporting Information. In supplementary methods, the mixed effects regression models are explained. In Supporting Information, the following tables and figures are available: Table S1: List of metabolites assigned and quantified in both serum and urine samples. Unknown metabolites are reported with ppm ranges and multiplicity. Table S2: Estimated average differences for urine metabolites between phase II and phase I (unique dosage). 90% confidence intervals are reported. Table S3: Estimated average differences for urine metabolites between consecutive samples (unique dosage). 90% confidence intervals are reported. Figure S1: Mixed effects models graphical representations. Models’ coefficients by rows, metabolites by columns. Each cell contains the estimated coefficient values colored by sign (positive or negative). Significant coefficients’ cells (*P*-value < 0.1) are red-framed. *R*^2^ index is reported for each model (the closer to 1, the better the model fit). (A) Urine metabolites with dosage A and B; (B) urine metabolites with unique dosage. Figure S2: Mixed effects models graphical representations for serum metabolites. Models’ coefficients by rows, serum metabolites by columns. Each cell contains the estimated coefficient values colored by sign (positive or negative). Significant coefficients’ cells (*P*-value < 0.1) are red- framed. An R-squared index is reported for each model (the closer to 1, the better the model fit). Figure S3: Average differences for urine metabolites between consecutive samples distinguishing for phase (type of line) and dosage group (color). Estimates and their 90% confidence intervals are colored by dosage group and the line type is different between phases. Figure S4: Average differences for urine metabolites between phase II and phase I. Estimates and their 90% confidence intervals are colored by dosage group. Figure S5: Average differences for urine metabolites between phase II and phase I (unique dosage). Figure S6: Average differences for urine metabolites between consecutive samples distinguishing for phase (type of line). Estimates and their 90% confidence intervals are reported. Figure S7: Average differences for serum metabolites between phase II and phase I. Estimates and their 90% confidence intervals are reported. All files are in.pdf extension (PDF)

## Abbreviations

1D: one-dimensional
AAs: amino acids
ATM: automatic tuning-matching
BCAAs: branched-chain amino acids
BMI: body mass index
CPMG: Carr–Purcell–Meiboom–Gill
MCCV: Monte Carlo cross-validation
mPLS-DA: multilevel partial least square discriminant analysis
NMR: nuclear magnetic resonance
NOESY: nuclear Overhauser effect spectroscopy
PCA: principal component analysis
PCA–CA–KNN: principal component analysis–canonical analysis–K-nearest neighbors
PQN: probabilistic quotient normalization
SOPs: standard operating procedures
TNF: tumor necrosis factor
