# Supplementary material for: Exploring the Effects of Probiotic Treatment on Urinary and Serum Metabolic Profiles in Healthy Individuals
Source: J Proteome Res. 2023 Nov 16;22(12):3866–78. doi: 10.1021/acs.jproteome.3c00548 (PMC10696601; doi:10.1021/acs.jproteome.3c00548)
Supplement: Supplementary file 1 — pr3c00548_si_001.pdf [file pr3c00548_si_001.pdf]

Exploring the effects of probiotic treatment on urinary and serum metabolic profiles in healthy individuals

Francesca Di Cesare [1, 2 ‡], *Matteo Calgaro* [3 ‡], *Veronica Ghini* [1, 2], *Diletta Francesca Squarzanti* [4,5], *Annachiara De Prisco* [6], *Annalisa Visciglia* [6], *Paola Zanetta* [4, 5], *Roberta Rolla* [4], *Paola Savoia* [4], *Angela Amoruso* [6], *Barbara Azzimonti* [4,5], *Nicola Vitulo* [3], *Leonardo Tenori* [1,2,7], *Claudio Luchinat* [7, 8*], *Marco Pane* [6*]

[1] Magnetic Resonance Center (CERM), , University of Florence, Via Luigi Sacconi 6, 50019, Sesto Fiorentino, Firenze, Italy

[2] Department of Chemistry "Ugo Schiff", University of Florence, Via della Lastruccia 3, 50019, Sesto Fiorentino, Italy

[3] Department of Biotechnology, University of Verona, Strada le Grazie, 15, 37134, Verona, Italy

[4] Department of Health Sciences (DiSS), University of Piemonte Orientale (UPO), Via Solaroli, 17, 28100, Novara, Italy

[5] Center for Translational Research on Autoimmune and Allergic Diseases (CAAD), Department of Health Sciences (DiSS), University of Piemonte Orientale (UPO), Corso Trieste, 15, 28100, Novara, Italy

[6] Probiotical Research Srl, Via Enrico Mattei, 3, 28100, Novara, Italy

[7] Consorzio Interuniversitario Risonanze Magnetiche MetalloProteine (CIRMMP), Via Luigi Sacconi 6, 50019, Sesto Fiorentino, Firenze, Italy

[8] Giotto Biotech S.r.l., Via Madonna del Piano, 6, 50019, Sesto Fiorentino, Firenze, Italy

[‡] Contributed equally

*To whom correspondence should be addressed: Claudio Luchinat, e-mail: luchinat@cerm.unifi.it; Marco Pane, e-mail: m.pane@probiotical.com

Table of Contents

Supplementary methods **3**
Urine metabolites - Dosage A and B . . . 3
Urine metabolites - Unique Dosage . . . 6
Serum metabolites . . . 8

Supplementary materials **10**
Tables . . . 10
Figures . . . 12

References **17**

Supplementary methods

Urine metabolites - Dosage A and B

As stated in the main text of the article, to study the urine metabolite trends and their relationship with the treatment, a mixed-effects linear regression framework is used for each metabolite S1. Using a simplified notation, the full model for the log-quantification of a generic urine metabolite is specified as it follows:

$$\begin{aligned}\log(Q) = {} & \mathit{Subject} + \mathit{Subject} \cdot \mathit{Sample} + \beta_0 + \beta_1 \mathit{Sample} + \beta_2(\mathit{Phase} = \mathit{II}) + \beta_3(\mathit{Dosage} = B) + \\ & + \beta_4 \mathit{Sample} : (\mathit{Phase} = \mathit{II}) + \beta_5(\mathit{Phase} = \mathit{II}) : (\mathit{Dosage} = B) + \\ & + \beta_6 \mathit{Sample} : (\mathit{Dosage} = B) + \beta_7 \mathit{Sample} : (\mathit{Phase} = \mathit{II}) : (\mathit{Dosage} = B) \\ & + \beta_8 \mathit{Age} + \beta_9(\mathit{Gender} = F) + \beta_{10} \mathit{BMI}\end{aligned}$$

Where:

- $\log(Q)$ is the dependent variable of the model, i.e. the log-quantification of a generic metabolite;
- $\mathit{Subject} + \mathit{Subject} \cdot \mathit{Sample}$ is the random part of the model: each **Subject** has a random intercept and slope, hence, for each subject, the trend in consecutive samples defined by the variable **Sample** (numerical, from 1 to 40, one for each consequent subject's measurement) could be different;
- β_0 is the fixed intercept;
- β_1 to β_7 are the coefficients for the **Sample** number, treatment **Dosage** (categorical, A or B), **Phase** (categorical, I for samples collected before the probiotic supplementation, II for samples collected during the probiotic supplementation), and their interactions;
- β_8 to β_{10} are the coefficients for **Age**, **Gender**, and **BMI** which were included in the models as they are considered possible confounding variables;
- the reference level for this model is represented by a male subject, belonging to dosage group A, before the treatment intake starts (*i.e.*, Phase I).

Estimate the models

To obtain a reduced model, more parsimonious than the full model, but with a comparable ability to describe the data variability, a stepwise model selection procedure is used. In detail, a log-likelihood ratio test is used to determine which variables are not significant for a given metabolite (p-value threshold = 0.1). In addition, two different models are estimated in each step of the selection: the first one with an undefined intra-group correlation structure between observations and the second model with an autoregressive correlation structure of the first order. In the latter model a relationship between consecutive observations is hypothesised (*e.g.*, sample 1 and sample 2, sample 2 and sample 3, etc.). The Akaike Information Criterion (AIC) is used to choose between the two models.

The R package `nlme` (v3.1-162)[1] is used to estimate the models.

Visualise the models

In Figure S1a every column represents a metabolite and each row represents a mixed-effects regression model coefficient (related to a covariate). Focusing on one metabolite, some of its cells could be empty, while others are colored. Empty cells indicates that the corresponding coefficients are not statistically significant in explaining the variability of that metabolite. On the contrary, colored cells contain the coefficient values which are somehow related to that metabolite. A red filled cell indicates a positive relationship between the coefficient variable and the metabolite, while a blue filled cell has the opposite meaning. A red square around the cell means that the coefficient associated p-value is below the threshold (p-value < 0.1). It may occur that a cell is filled without the red square around it. This is the case when the interaction terms are

significant for a metabolite. Indeed, the main effects are kept into the model even if their p-values are not significant when the interactions are significant.

Regarding the interpretability of each model:

- the main effects indicate an average difference between the level in the coefficient row (*e.g.*, Phase = II) and its reference level (*e.g.*, Phase = I);
- the Sample covariate indicates the estimated increase/decrease of the corresponding metabolite values when the Sample changes by a single unit (*i.e.*, the average difference between consequent samples);
- the interaction terms measure metabolite changes in presence of one or more conditions compared to the baseline levels (Phase = I, Dosage = “A”).

Since all the subjects are treated in Phase II, the Phase covariate is directly linked to the probiotic supplementation effect. A positive or negative value for the coefficient of Phase = II is related to an increase or decrease of the average log-quantification level for the corresponding metabolite compared to Phase I. However, it cannot be considered by its own when interactions are significant:

- when the coefficient for the interaction between Phase and Dosage is significant, an average difference of log-quantification values is present for the corresponding metabolite and it is not the same across phases and dosage groups;
- when the coefficient for the interaction between Sample and Phase is significant, an increasing or decreasing trend is present for the corresponding metabolite and it is not the same in Phase I and Phase II;
- when the coefficient for the interaction between Sample and Dosage is significant, an increasing or decreasing trend is present for the corresponding metabolite and it is not the same in dosage groups;
- when the coefficient for the interaction between Sample, Phase, and Dosage is significant, the previous trends could be different between phases and also between dosage groups.

The determination index R^2 measures the amount of variability explained by each model (computed using the `MuMIn` package v1.47.5[2]).

Extract the results

To describe metabolic variations across conditions the estimated models can be used. Some “contrasts” of interest are evaluated, using the `multcomp` R package v1.4-23[3], in order to answer biologically relevant questions:

- Is the average level of the metabolite the same in Phase I and Phase II, net of other variables?
- Is the average level of the metabolite stable between consecutive samples or is there an increasing/decreasing trend?

Estimated average differences between Phase II and Phase I To measure the average difference between Phase II and Phase I, for a given metabolite, estimated log quantification levels in the middle of each phase are compared. According to the experimental design, while the 10th sample is in the middle of the Phase I, the 30th sample is not in the middle of the Phase II. Indeed, the first sample of the Phase II is taken after 28 days of probiotics supplementation. However, as 20 samples are collected for each phase, the 30th sample is, numerically speaking, the sample in middle of the collected samples of Phase II. From a statistical perspective, the regression estimates of these samples are exactly in the middle of the two phases representing a meaningful choice to summarize them. Indeed it is robust with respect to the phase trends which may occur when a metabolite is increasing and/or decreasing during one or both phases.

The following null hypothesis is tested:

> The average level of the metabolite is the same in Phase I and Phase II, net of other variables.

It can be translated using the regression model formulation:

- In dosage group A:

$$\begin{aligned}
M_{30_A} &= \beta_0 + \beta_1(Sample = 30) + \beta_2(Phase = II) + \beta_4(Sample = 30) : (Phase = II)) \\
M_{10_A} &= \beta_0 + \beta_1(Sample = 10)) \\
M_{30_A} - M_{10_A} &= \beta_1(30 - 10) + \beta_2 + \beta_4(30) = 0
\end{aligned}$$

Which corresponds to $H_0 : 20\beta_1 + \beta_2 + 30\beta_4 = 0$

- In dosage group B:

$$\begin{aligned}
M_{30_B} = \beta_0 &+ \beta_1(Sample = 30) + \beta_2(Phase = II) + \beta_3(Dosage = B) + \beta_4(Sample = 30) : (Phase = II)+ \\
&+ \beta_5(Phase = II) : (Dosage = B) + \beta_6(Sample = 30) : (Dosage = B)+ \\
&+ \beta_7(Sample = 30) : (Phase = II) : (Dosage = B)- \\
M_{10_B} &= \beta_0 + \beta_1(Sample = 10) + \beta_3(Dosage = B) + \beta_6(Sample = 10) : (Dosage = B)) \\
M_{30_B} - M_{10_B} &= \beta_1(30 - 10) + \beta_2 + \beta_4(30) + \beta_5 + \beta_6(30 - 10) + \beta_7(30) = 0
\end{aligned}$$

Which corresponds to $H_0 : 20\beta_1 + \beta_2 + 30\beta_4 + \beta_5 + 20\beta_6 + 30\beta_7 = 0$

The statistically significant differences are reported in Table 4 of the main text. The same results are also summarized in Figure S4.

Average differences between consequent samples The choice to compare samples in different phases allows to describe differences between phases. However, the stability of a metabolite within the 20 samples of each phase can also be investigated. This can be done by comparing consecutive samples.

The following null hypothesis is tested:

The average level of the metabolite is the same between consecutive samples, net of other variables.

It can be translated using the regression model formulation:

- In dosage group A during Phase I:

$$\begin{aligned}
M_{2_A} &= \beta_0 + \beta_1(Sample = 2) \\
M_{1_A} &= \beta_0 + \beta_1(Sample = 1) \\
M_{2_A} - M_{1_A} &= \beta_1(2 - 1) = 0
\end{aligned}$$

Which corresponds to $H_0 : \beta_1 = 0$

- In dosage group B during Phase I:

$$\begin{aligned}
M_{2_B} &= \beta_0 + \beta_1(Sample = 2) + \beta_3(Dosage = B) + \beta_6(Sample = 2) : (Dosage = B) \\
M_{1_B} &= \beta_0 + \beta_1(Sample = 1) + \beta_3(Dosage = B) + \beta_6(Sample = 1) : (Dosage = B)) \\
M_{2_B} - M_{1_B} &= \beta_1(2 - 1) + \beta_6(2 - 1) = 0
\end{aligned}$$

Which corresponds to $H_0 : \beta_1 + \beta_6 = 0$

- In dosage group A during Phase II:

$$
\begin{aligned}
M_{22_A} &= \beta_0 + \beta_1(Sample = 22) + \beta_2(Phase = II) + \beta_4(Sample = 22) : (Phase = II) \\
M_{21_A} &= \beta_0 + \beta_1(Sample = 21) + \beta_2(Phase = II) + \beta_4(Sample = 21) : (Phase = II) \\
M_{22_A} - M_{21_A} &= \beta_1(22 - 21) + \beta_4(22 - 21) = 0
\end{aligned}
$$

Which corresponds to $H_0 : \beta_1 + \beta_4 = 0$

- In dosage group B during Phase II:

$$
\begin{aligned}
M_{22_B} &= \beta_0 + \beta_1(Sample = 22) + \beta_2(Phase = II) + \beta_3(Dosage = B)+ \\
&\quad + \beta_4(Sample = 22) : (Phase = II) + \beta_5(Phase = II) : (Dosage = B)+ \\
&\quad + \beta_6(Sample = 22) : (Dosage = B) + \beta_7(Sample = 22) : (Phase = II) : (Dosage = B) \\
M_{21_B} &= \beta_0 + \beta_1(Sample = 21) + \beta_2(Phase = II) + \beta_3(Dosage = B)+ \\
&\quad + \beta_4(Sample = 21) : (Phase = II) + \beta_5(Phase = II) : (Dosage = B)+ \\
&\quad + \beta_6(Sample = 21) : (Dosage = B) + \beta_7(Sample = 21) : (Phase = II) : (Dosage = B) \\
M_{22_B} - M_{21_B} &= \beta_1(22 - 21) + \beta_4(22 - 21) + \beta_6(22 - 21) + \beta_7(22 - 21) = 0
\end{aligned}
$$

Which corresponds to $H_0 : \beta_1 + \beta_4 + \beta_6 + \beta_7 = 0$

The statistically significant differences are reported in Table 3 of the main text. The same results are also summarized in Figure S3. Differences above zero indicate an increase of the average log-quantification values of the corresponding metabolites between consecutive samples (a positive trend). On the contrary, all the estimates below the zero line, indicate a reduction of the corresponding metabolites' log-quantification values (a negative trend). The higher the absolute value of the estimate, the bigger the difference. Instead, for the 90% confidence interval bars, the narrower they are, the smaller the variability of the estimated difference is. Colors and line types represent the different dosage groups and the phase respectively. Indeed, each metabolite can be characterized by a positive trend in one phase (or one dosage group) and a different behavior in the other group.

Urine metabolites - Unique Dosage

Assuming the equality of the effects for the two dosage groups over time translates to a simplified version of the mixed-effects linear regression model. The Dosage variable and its interactions with Sample and Phase are no longer included in the models. Using a simplified notation, the full model for the log-quantification of a generic urine metabolite is specified as it follows:

$$
\begin{aligned}
\log(Q) = Subject + Subject \cdot Sample + \beta_0 + \beta_1 Sample + \beta_2(Phase = II)+ \\
+ \beta_3 Sample : (Phase = II) + \beta_4 Age + \beta_5(Gender = F) + \beta_6 BMI
\end{aligned}
$$

Where:

- $\log(Q)$ is the dependent variable of the model, i.e. the log-quantification of a generic metabolite;
- $Subject + Subject \cdot Sample$ is the random part of the model: each **Subject** has a random intercept and slope, hence, for each subject, the trend in consecutive samples defined by the variable **Sample** (numerical, from 1 to 40, one for each consequent subject's measurement) could be different;
- β_0 is the fixed intercept;
- β_1 to β_3 are the coefficients for the **Sample** number, **Phase** (categorical, I for samples collected before the probiotic supplementation, II for samples collected during the probiotic supplementation), and their interaction;

- β_4 to β_6 are the coefficients for **Age**, **Gender**, and **BMI** which were included in the models as they are considered possible confounding variables;
- the reference level for this model is represented by a male subject, before the treatment intake starts (*i.e.*, Phase I).

Estimate the models

As in 1.1.1, to obtain a reduced model, more parsimonious than the full model, but with a comparable ability to describe the data variability, a stepwise model selection procedure is used.

Visualise the models

In Figure S1b the mixed-effects regression model results are presented for each urine metabolite.

Regarding the interpretability of each model:

- the main effects indicate an average difference between the level in the coefficient row (*e.g.*, Phase = II) and its reference level (*e.g.*, Phase = I);
- the Sample covariate indicates the estimated increase/decrease of the corresponding metabolite values when the Sample changes by a single unit (*i.e.*, the average difference between consequent samples);
- the interaction term measures metabolite changes in presence of one or more conditions compared to the baseline levels.

Extract the results

To describe metabolic variations across conditions the estimated models can be used. Some "contrasts" of interest are evaluated in order to answer biologically relevant questions:

- Is the estimated average level of the metabolite the same in Phase I and Phase II, net of other variables?
- Is the average level of the metabolite stable between consecutive samples or is there an increasing/decreasing trend?

Estimated average differences between Phase II and Phase I As in 1.1.3.1, to measure the average difference between Phase II and Phase I, for a given metabolite, estimated log quantification levels in the middle of each phase are compared.

The following null hypothesis is tested:

The average level of the metabolite is the same in Phase I and Phase II, net of other variables.

It can be translated using the regression model formulation:

$$M_{30} = \beta_0 + \beta_1(Sample = 30) + \beta_2(Phase = II) + \beta_3(Sample = 30) : (Phase = II))$$
$$M_{10} = \beta_0 + \beta_1(Sample = 10))$$
$$M_{30} - M_{10_A} = \beta_1(30 - 10) + \beta_2 + \beta_3(30) = 0$$

Which corresponds to $H_0 : 20\beta_1 + \beta_2 + 30\beta_3 = 0$

The statistically significant differences are reported in Table S2. The same results are also summarized in Figure S5.

Average differences between consequent samples Similarly to the analysis performed in 1.1.3.2, the stability of a metabolite within the 20 samples of each phase is investigated by comparing consecutive samples.

The following null hypothesis is tested:

> The average level of the metabolite is the same between consecutive samples, net of other variables.

It can be translated using the regression model formulation:

- During Phase I:

$$\begin{aligned} M_2 &= \beta_0 + \beta_1(Sample = 2) \\ M_1 &= \beta_0 + \beta_1(Sample = 1) \\ M_2 - M_1 &= \beta_1(2 - 1) = 0 \end{aligned}$$

  Which corresponds to $H_0 : \beta_1 = 0$

- During Phase II:

$$\begin{aligned} M_{22} &= \beta_0 + \beta_1(Sample = 22) + \beta_2(Phase = II) + \beta_3(Sample = 22) : (Phase = II) \\ M_{21} &= \beta_0 + \beta_1(Sample = 21) + \beta_2(Phase = II) + \beta_3(Sample = 21) : (Phase = II) \\ M_{22} - M_{21} &= \beta_1(22 - 21) + \beta_3(22 - 21) = 0 \end{aligned}$$

  Which corresponds to $H_0 : \beta_1 + \beta_3 = 0$

The statistically significant differences are reported in Table S3. The same results are also summarized in Figure S6.

Serum metabolites

To study the serum metabolite average levels and their relationship with the treatment, a mixed-effects linear regression framework is used for each metabolite S1. While 20 urine samples were collected for each phase, serum samples are only 2, the first is collected at the beginning of Phase I and the other at the beginning of Phase II. As stated in the main text, the model formulation for the log-quantification of a generic serum metabolite is much simpler than the one for urine metabolites and it is specified as it follows:

$$\begin{aligned} \log(Q) = {} & Subject + \beta_0 + \beta_1(Phase = II) + \beta_2(Dosage = B) + \beta_4(Phase = II) : (Dosage = B) + \\ & + \beta_5 Age + \beta_6(Gender = F) + \beta_7 BMI \end{aligned}$$

Where:

- $\log(Q)$ is the dependent variable of the model, i.e. the log-quantification of a generic metabolite;
- *Subject* is the random part of the model: each **Subject** has a random intercept;
- β_0 is the fixed intercept;
- β_1 to β_4 are the coefficients for the **Dosage** group (categorical, A or B), **Phase** (categorical, I for the sample collected before the probiotic supplementation, II for the sample collected during the probiotic supplementation), and their interactions;
- β_5 to β_7 are the coefficients for **Age**, **Gender**, and **BMI** which were included in the models as they are considered possible confounding variables;
- the reference level for this model is represented by a male subject, belonging to dosage group A, before the treatment intake starts (*i.e.*, Phase I).

Estimate the models

As for the urine metabolites' models, to obtain reduced models, more parsimonious than the full models, but with a comparable ability to describe the data variability, a stepwise model selection procedure is used.

Visualise the models

In Figure S2 the mixed-effects regression model results are presented for each serum metabolite.

Regarding the interpretability of each model:

- the main effects indicate an average difference between the level in the coefficient row (*e.g.*, Phase = II) and its reference level (*e.g.*, Phase = I);
- the interaction term between Phase and Dosage is never significant. Hence, differences between phases are present but they are not different between dosage groups.

Among the other covariates included in the models, both Age, Gender, and BMI are able to explain metabolite levels (*e.g.*, when the variable Gender is significant, we observe a reduction for the metabolite levels in females).

Extract the results

To describe metabolic variations across conditions the estimated models can be used. Some "contrasts" of interest are evaluated in order to answer a biologically relevant question:

- Is the average level of the metabolite the same in Phase I and Phase II, net of other variables?

Estimated average differences between Phase I and Phase II The choice to compare the sample collected in Phase I and the sample collected in Phase II is given by the need to capture differences between phases. Chronologically speaking, the Phase I sample was collected at the beginning of the Phase I, instead the Phase II sample was collected after 28 days of probiotics supplementation.

The following null hypothesis is tested:

> The average level of the metabolite is the same in both phases, net of other variables.

It can be translated using the regression model formulation:

$$\begin{aligned} M_{Phase_{II}} &= \beta_0 + \beta_1(Phase = II) \\ M_{Phase_I} &= \beta_0 \\ M_{Phase_{II}} - M_{Phase_I} &= \beta_1 = 0 \end{aligned}$$

Which corresponds to $H_0 : \beta_1 = 0$

The statistically significant differences are reported in Table 5 of the main text. The same results are also summarized in Figure S7.

All serum metabolites were not changing differently by dosage group between phases (no significant Dosage-Phase interactions). For this reason the unique dosage analysis is not performed for serum metabolites.

Supplementary materials

Tables

Table S1: List of metabolites assigned and quantified in both serum and urine samples. Unknown metabolites are reported with ppm ranges and multiplicity.

**Serum metabolites**	
Acetate	Histidine
Acetone	Isoleucine
Alanine	Lactate
Betaine	Leucine
Citrate 1 (ppm range = 2.559 - 2.545, singlet 1)	Lysine
Citrate 2 (ppm range = 2.571 - 2.560, singlet 2)	Mannose
Creatine	Phenylalanine
Creatinine	Proline
Formate	Pyruvate
Glycoprotein	Threonine
Glucose 1 (ppm range = 5.252 - 5.230, doublet)	Tyrosine
Glucose 2 (ppm range = 3.542 - 3.525, singlet)	Valine
Glutamine	Unk1 (ppm range = 1.072 - 1.063, singlet)
Glycine	Unk2 (ppm range = 1.1210 - 1.110, singlet)
**Urine metabolites**	
Acetoacetic Acid	Leucine
Acetone	Lysine
Alanine	Phenylacetylglutamine
Allantoin	Tartrate
Asparagine	Trimethylamine-N-oxide (TMAO)
Citrate	Trigonelline
Creatine + Creatinine	Tyrosine
Creatinine	Valine
Dimethylamine	1-methylnicotinamide
dTTP	2-hydroxyisobutyric acid
Formate	3-hydroxyisobutyric acid
Fumarate	3-hydroxymandelate
Glycine	3-hydroxypropanoic acid (HPHPA)
Glucose	4-hydroxyphenylacetate
Hippurate	Unk1 (ppm range = 0.940 - 0.932, singlet)
Histidine	Unk2 (ppm range = 2.910 - 2.886, singlet)
Isoleucine	Unk3 (ppm range = 2.790 - 2.781, singlet)
Indoxyl-sulphate	Unk4 (ppm range = 5.410 - 5.400, singlet)
Lactic acid + Threonine	Sugar Unk (ppm range = 5.218 - 5.200)

Table S2: Estimated average differences for urine metabolites between Phase II and Phase I (unique dosage). 90% confidence intervals are reported.

		90% Confidence Interval	
**Metabolite**	**Difference**	**Lower**	**Upper**
Tartrate	0.2295419	0.1107101	0.3483737
Isoleucine	-0.0261602	-0.0453173	-0.0070032
Valine	-0.0292876	-0.0479422	-0.0106330
X3hydroxyisobutyricAcid	-0.0399144	-0.0697436	-0.0100853
Unk4	-0.0463896	-0.0799924	-0.0127868
Allantoin	-0.0484812	-0.0850020	-0.0119604
X4hydroxyphenylacetate	-0.0497978	-0.0834943	-0.0161012
Glucose	-0.1271036	-0.1739512	-0.0802560

Table S3: Estimated average differences for urine metabolites between consecutive samples (unique dosage). 90% confidence intervals are reported.

		90% Confidence Interval		
**Metabolite**	**Difference**	**Lower**	**Upper**	**Phase**
Hippurate	0.0101485	0.0025794	0.0177177	I
X2hydroxyisobutyricAcid	0.0023444	0.0001042	0.0045846	I
Phenylacetylglutamine	-0.0031162	-0.0059046	-0.0003278	I
SugarUnk	-0.0032036	-0.0061667	-0.0002406	I
X4hydroxyphenylacetate	-0.0037069	-0.0073998	-0.0000139	I
Glucose	-0.0057321	-0.0100947	-0.0013695	I
AcetoaceticAcid	-0.0066291	-0.0120203	-0.0012378	I
Formate	0.0045674	0.0008955	0.0082393	I and II
AcetoaceticAcid	0.0066941	0.0013029	0.0120853	II
Glucose	0.0044161	0.0000535	0.0087787	II
SugarUnk	0.0031748	0.0002118	0.0061378	II
Lysine	-0.0024063	-0.0044503	-0.0003623	II
TMAO	-0.0151342	-0.0240711	-0.0061972	II

Figures

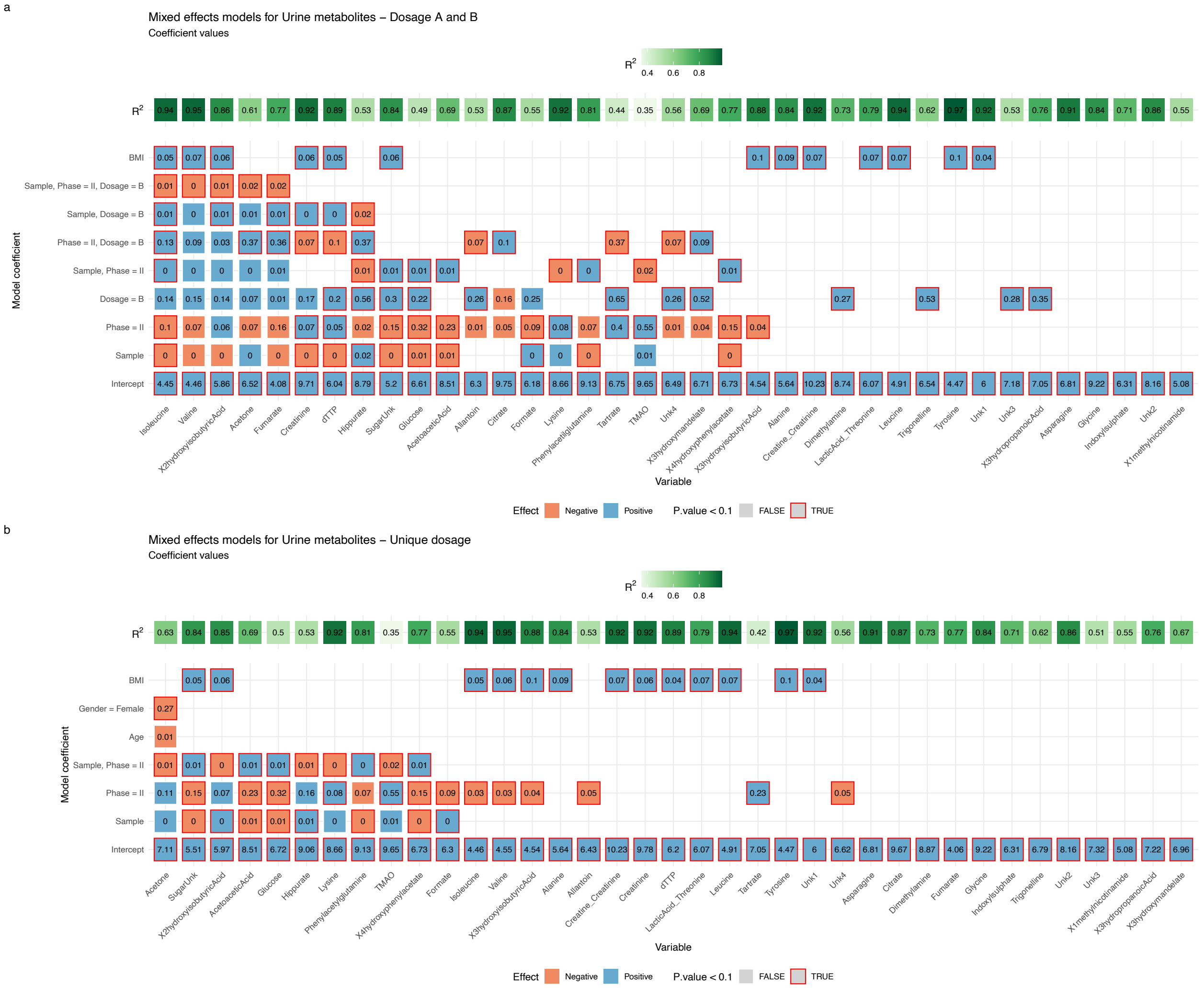

Figure S1: Mixed effects models graphical representations. Models' coefficients by rows, metabolites by columns. Each cell contains the estimated coefficient values colored by sign (positive or negative). Significant coefficients' cells (p-value < 0.1) are red-framed. R-squared index is reported for each model (the closer to 1, the better the model fit). **a.** Urine metabolites with Dosage A and B. **b.** Urine metabolites with unique dosage.

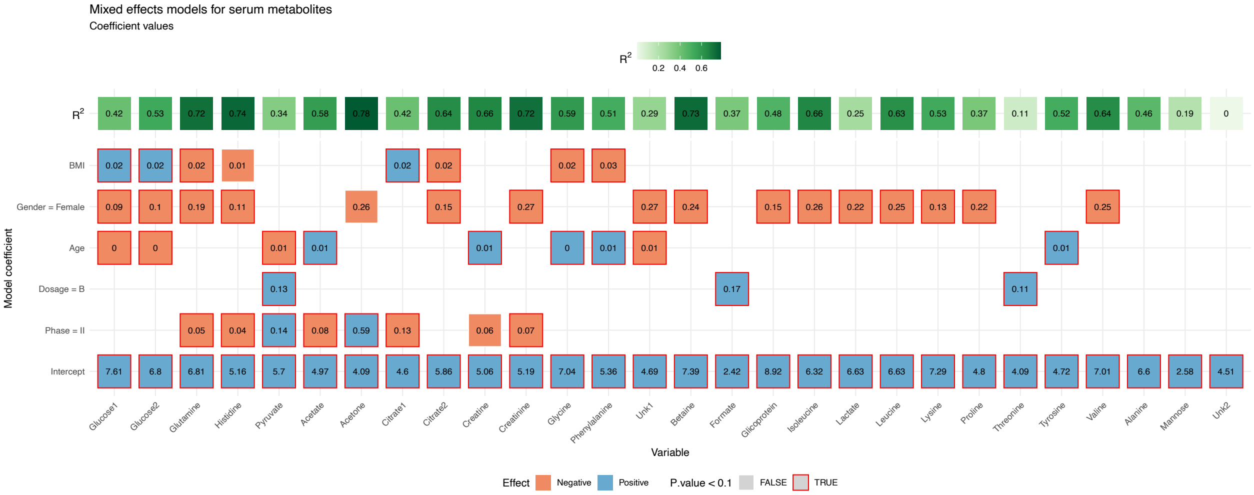

Figure S2: Mixed effects models graphical representations for serum metabolites. Models' coefficients by rows, serum metabolites by columns. Each cell contains the estimated coefficient values colored by sign (positive or negative). Significant coefficients' cells (p-value < 0.1) are red-framed. R-squared index is reported for each model (the closer to 1, the better the model fit).

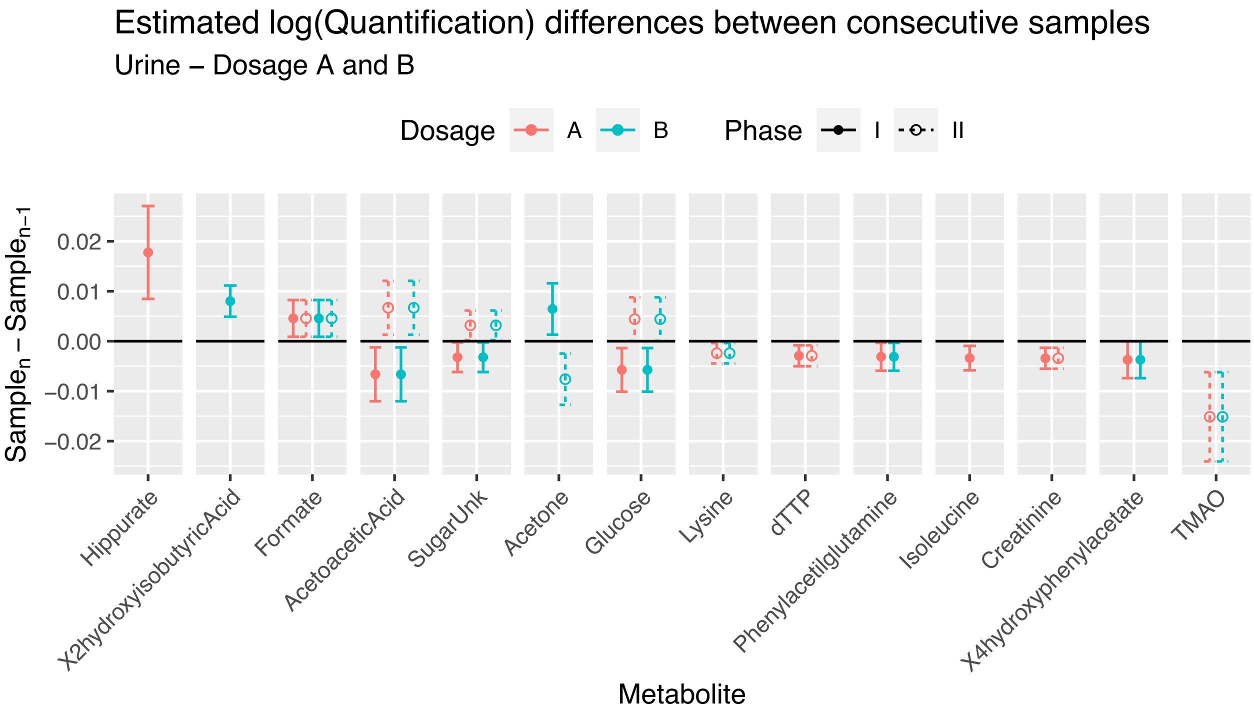

Figure S3: Average differences for urine metabolites between consecutive samples distinguishing for Phase (type of line) and Dosage group (color). Estimates and their 90% confidence intervals are colored by dosage group and the line type is different between phases.

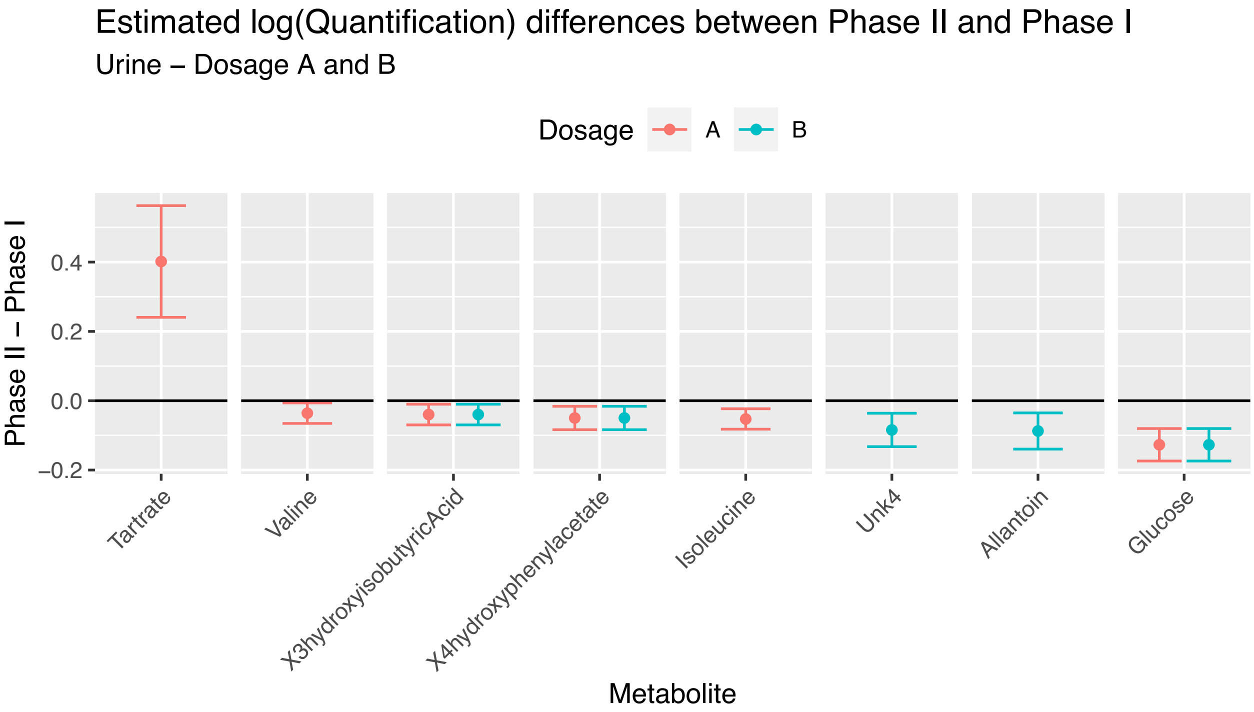

Figure S4: Average differences for urine metabolites between Phase II and Phase I. Estimates and their 90% confidence intervals are colored by dosage group.

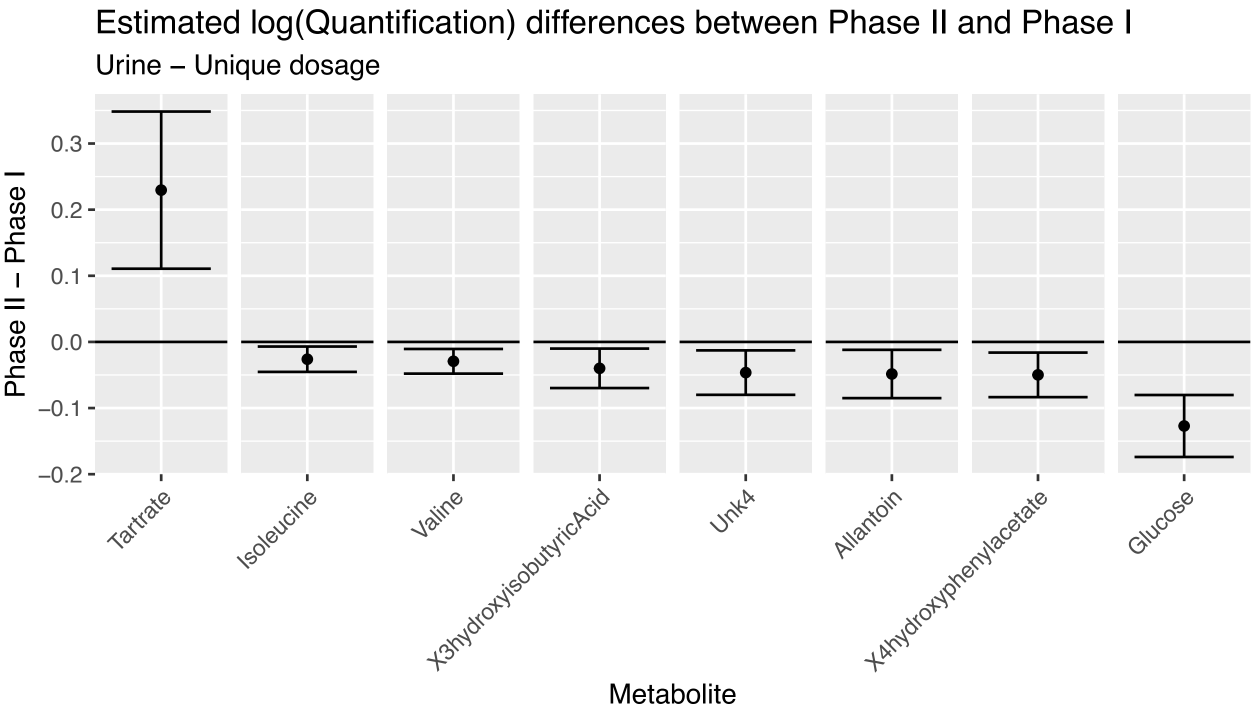

Figure S5: Average differences for urine metabolites between Phase II and Phase I (unique dosage).

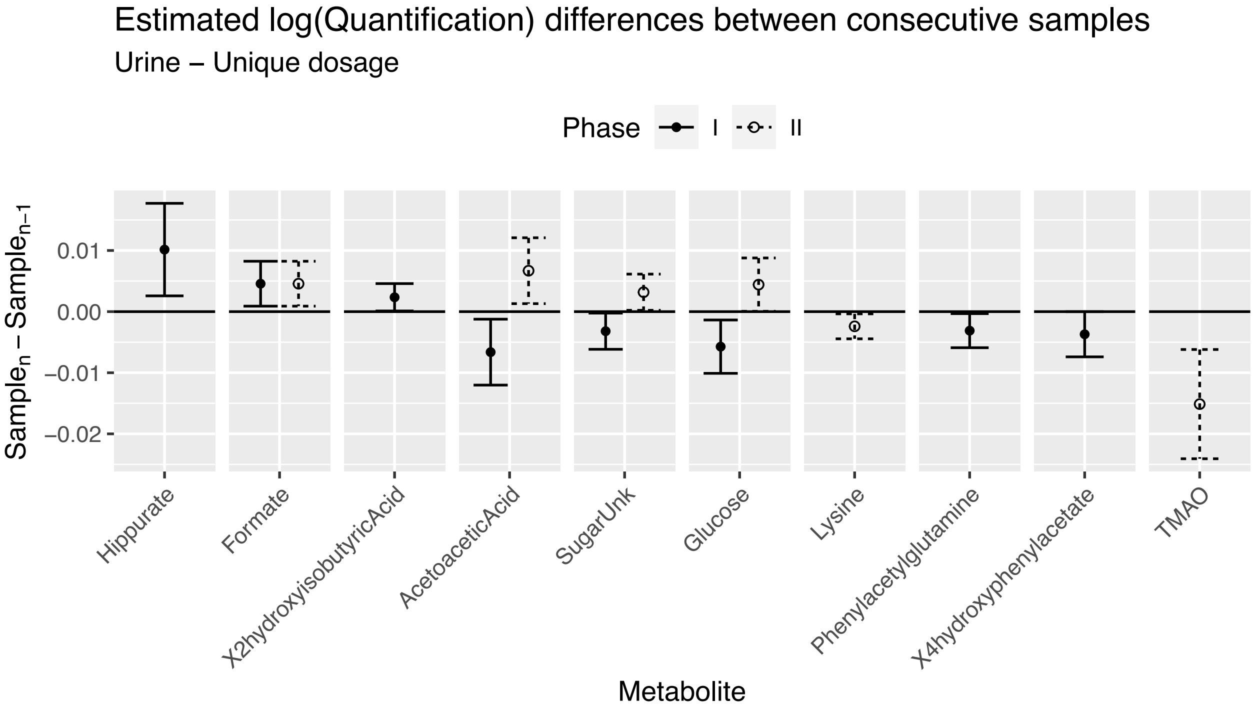

Figure S6: Average differences for urine metabolites between consecutive samples distinguishing for Phase (type of line). Estimates and their 90% confidence intervals are reported.

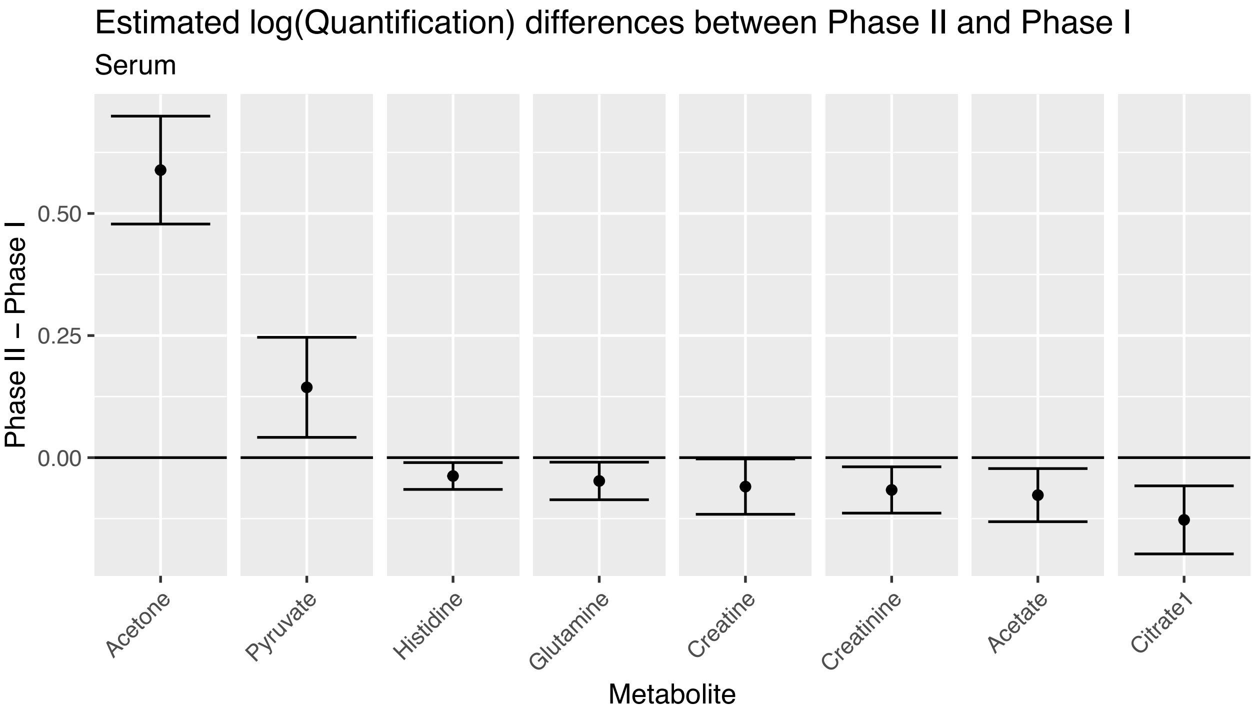

Figure S7: Average differences for serum metabolites between Phase II Phase I. Estimates and their 90% confidence intervals are reported.

References

(1) Pinheiro J, Bates D, R Core Team (2023). nlme: Linear and Nonlinear Mixed Effects Models. R package version 3.1-162, https://CRAN.R-project.org/package=nlme.

(2) Bartoń K (2023). MuMIn: Multi-Model Inference. R package version 1.47.5, https://CRAN.R-project.org/package=MuMIn.

(3) Hothorn, T.; Bretz, F.; Westfall, P. Simultaneous Inference in General Parametric Models. *Biometrical Journal* **2008**, *50* (3), 346–363. https://doi.org/10.1002/bimj.200810425.
